# P-1456. Evaluating the Successes & Limitations of the University of Rochester Medical Center COVID-19 Monoclonal Antibody Program

**DOI:** 10.1093/ofid/ofae631.1628

**Published:** 2025-01-29

**Authors:** Troy J Anlage, Calvin Albrecht, Mike Sportiello, Christine Kim, Sage Hellerstedt, Zhongmou Sun, Genevieve Medina, Ellie Camanzo, Ted Louie

**Affiliations:** University of Rochester Medical Center, Rochester, New York; University of Rochester Medical Center, Rochester, New York; University of Rochester Medical Center, Rochester, New York; University of Rochester Medical Center, Rochester, New York; University of Rochester, Rochester, New York; University of Rochester School of Medicine and Dentistry, Rochester, New York; University of Rochester Medical Center, Rochester, New York; University of Rochester Medical Center, Rochester, New York; University of Rochester Medical Center, Rochester, New York

## Abstract

**Background:**

The rapid spread of SARS-CoV-2 was particularly impactful in underserved communities, including in Monroe County, NY, which is medium-high on the Social Vulnerability Index (SVI). To combat rising COVID-19 mortality, the FDA authorized monoclonal antibodies (mAbs) under Emergency Use Authorization for treatment, and later for prophylaxis against COVID-19 in high-risk individuals. At the University of Rochester Medical Center (URMC), programs were developed to administer prophylactic and treatment mAbs to select patients at high risk for severe COVID-19. The purpose of this study was to: 1) determine the clinical outcomes of patients receiving prophylactic or therapeutic mAbs stratified by SVI, and 2) assess the equitability of mAb allocation at URMC.

Indications for COVID-19 mAb Prophylaxis

**Figure:**
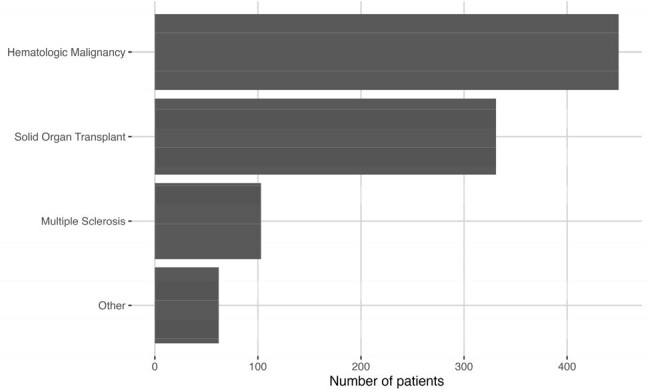

**Methods:**

We performed a single-center retrospective study of over 1,300 adult patients who received prophylactic or treatment mAbs at select URMC outpatient clinics or in the emergency department. Stratification of socioeconomic status was achieved using SVI, with higher scores reflecting greater vulnerability. SVI was determined for each patient using publicly available CDC data. A chart review was completed to determine the qualifying diagnosis for prophylactic mAbs, COVID-19 testing status within 6 months of receiving prophylactic mAbs, hospitalization rates, and COVID-19 disease severity. Data analysis was performed in R v4.3.2 using rstatix_0.7.2.

SARS-CoV-2 Positivity Within 6 Months of Receiving Prophylactic mAb

**Figure:**
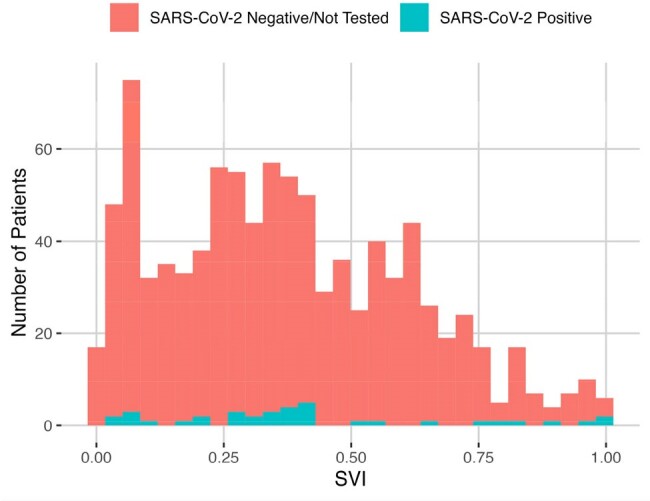

Lower SVI (0.00) indicates lower social vulnerability while higher SVI (1.00) indicates higher social vulnerability.

**Results:**

Higher SVI was strongly associated with infection (P=0.0075, OR=3.82) and hospitalizations (P=0.033, OR=5.10) attributed to SARS-CoV-2 in those who received **prophylactic** mAbs. Among patients who received **treatment** mAbs, SVI was significantly higher (P=0.011 by Wilcoxon rank-sum test) for patients hospitalized for COVID-19 versus those who were not hospitalized.

COVID-19 Hospitalizations Within 6 Months of Receiving Treatment mAb

**Figure:**
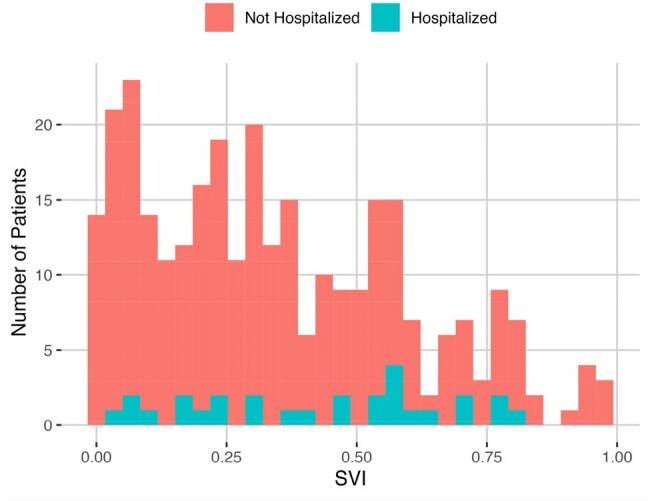

Lower SVI (0.00) indicates lower social vulnerability while higher SVI (1.00) indicates higher social vulnerability.

**Conclusion:**

Patients with higher SVI who received prophylactic or treatment mAbs experienced worse COVID-19 clinical outcomes. Further studies designed to address allocation are needed to determine if patients with higher SVI received a disproportionately smaller share of mAbs. Priority should be given to patients with higher SVI in future programs requiring distribution of novel emergency therapies to address these disparities.

SARS-CoV-2 Positivity Within 6 Months of Receiving Prophylactic mAb by Prophylaxis Indication

**Figure:**
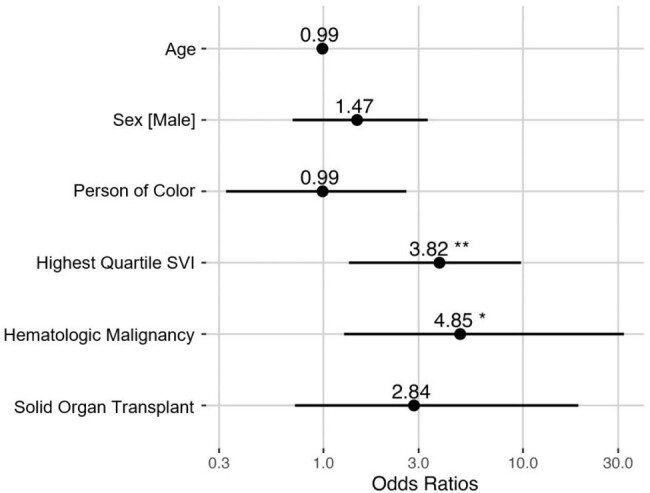

Asterisks (* p < 0.05, ** p < 0.01) indicate statistical significance in logistic regression model.

**Disclosures:**

**All Authors**: No reported disclosures

